# *Harmonia axyridis* (Coleoptera: Coccinellidae) Exhibits No Preference between Bt and Non-Bt Maize Fed *Spodoptera frugiperda* (Lepidoptera: Noctuidae)

**DOI:** 10.1371/journal.pone.0044867

**Published:** 2012-09-14

**Authors:** Carla C. Dutra, Robert L. Koch, Eric C. Burkness, Michael Meissle, Joerg Romeis, William D. Hutchison, Marcos G. Fernandes

**Affiliations:** 1 Faculdade de Ciências Agrárias, Universidade Federal da Grande Dourados, Dourados, Brazil; 2 Plant Protection Division, Minnesota Department of Agriculture, Saint Paul, Minnesota, United States of America; 3 Department of Entomology, University of Minnesota, St. Paul, Minnesota, United States of America; 4 Agroscope Reckenholz-Tänikon Research Station ART, Zurich, Switzerland; 5 Faculdade de Ciências Biológicas e Ambientais, Universidade Federal da Grande Dourados, Dourados, Brazil; University of Kentucky, United States of America

## Abstract

A recent shift in managing insect resistance to genetically engineered (GE) maize consists of mixing non-GE seed with GE seed known as “refuge in a bag”, which increases the likelihood of predators encountering both prey fed Bt and prey fed non-Bt maize. We therefore conducted laboratory choice-test feeding studies to determine if a predator, *Harmonia axyridis*, shows any preference between prey fed Bt and non-Bt maize leaves. The prey species was *Spodoptera frugiperda*, which were fed Bt maize (MON-810), expressing the single Cry1Ab protein, or non-Bt maize. The predators were third instar larvae and female adults of *H. axyridis*. Individual predators were offered Bt and non-Bt fed prey larvae that had fed for 24, 48 or 72 h. Ten and 15 larvae of each prey type were offered to third instar and adult predators, respectively. Observations of arenas were conducted at 1, 2, 3, 6, 15 and 24 h after the start of the experiment to determine the number and type of prey eaten by each individual predator. Prey larvae that fed on non-Bt leaves were significantly larger than larvae fed Bt leaves. Both predator stages had eaten nearly all the prey by the end of the experiment. However, in all combinations of predator stage and prey age, the number of each prey type consumed did not differ significantly. ELISA measurements confirmed the presence of Cry1Ab in leaf tissue (23–33 µg/g dry weight) and *S. frugiperda* (2.1–2.2 µg/g), while mean concentrations in *H. axyridis* were very low (0.01–0.2 µg/g). These results confirm the predatory status of *H. axyridis* on *S. frugiperda* and that both *H. axyridis adults and larvae* show no preference between prey types. The lack of preference between Bt-fed and non-Bt-fed prey should act in favor of insect resistance management strategies using mixtures of GE and non-GE maize seed.

## Introduction

Genetically engineered (GE) plants, modified to resist insect pests via expression of various *Bacillus thuringiensis* Berliner (Bt) toxins, have been successfully commercialized in several countries [1]. Since 1996, GE maize (*Zea mays* L.) has been the most widely grown Bt crop in the U.S., primarily due to its efficacy against the European corn borer, *Ostrinia nubilalis* (Hübner) (Lepidoptera: Crambidae) [2], [3]. Recent approvals for Bt maize in Brazil suggest adoption rates among growers will also be high, because of the widespread damage caused by Lepidoptera pests such as the fall armyworm, *Spodoptera frugiperda* (JE Smith) (Lepidoptera: Noctuidae), a pest responsible for significant losses (17 to 39%) in maize production [4]–[7]. Since commercialization of Bt maize in 2008, Brazil has increased its rate of biotech crop production more than any other country worldwide; a record 4.9 million ha increase, equivalent to an annual increase of 20% [1].

The focus of the present study was to assess the risk of potential adverse effects of GE maize on non-target arthropods. Previous studies have shown that Cry proteins of Bt expressed in GM plants can be acquired by non-target herbivores and predators, but in tri-trophic interactions, herbivore consumption of Cry proteins has not negatively affected the survival or other life history parameters of the third trophic level, such as heteropterans [8], [9] and lady beetles [10]. In addition, consideration must be given to insect resistance management (IRM). As the use of GE maize expands and the technology of transgenic crops evolves, IRM must also evolve to provide sustainable crop pest management [11], [12].

To mitigate the risk of Bt resistance evolution in insect pests, the U.S. Environmental Protection Agency has required farmers to plant non-Bt maize refuge areas. For *O. nubilalis* in the U.S., for example, non-Bt maize refuges had to be planted within 0.5 miles of the Bt maize [13]. In recent years, primarily due to the concerns of resistance in the western corn rootworm, *Diabrotica virgifera virgifera* (LeConte) (Coleoptera: Chrysomelidae), a new refuge strategy was developed consisting of a mixture of Bt and non-Bt seed resulting in a random mix of non-Bt plants within a Bt maize field [11]. This mixture of Bt seed with low proportions of non-Bt seed in seed bags or in planters (e.g., 5–10% non-GE) is referred to as “refuge in a bag” [11]. By integrating pests more evenly throughout a field, seed mixtures may facilitate the persistence of natural enemies within maize fields better than the current refuge strategy [11], [14], with a potential impact on resistance development [15]. In such a setting, the co-occurrence of Bt-fed and non-Bt-fed herbivores will be greater than ever, increasing the likelihood that predators in the field will actually be confronted with a choice between Bt-fed and non-Bt-fed prey.

In Brazil, the intensity of damage caused by fall armyworm is high when natural enemies are not present in the field [16]. Entomophagous lady beetles have frequently been reported to prey on eggs and larvae of some species of Lepidoptera, including the fall armyworm [17]. In South America, an invasion of a predatory coccinellid, *Harmonia axyridis* (Pallas) (Coleoptera: Coccinellidae), is underway [18], [19]. Established populations were first detected in Argentina in 2001 and in Brazil in 2002 [20] and has now been detected in at least seven Brazilian states [19]. This coccinellid occurs in many agricultural systems where it feeds primarily on aphids, but is also known to prey on other arthropods including immature Lepidoptera [21]. Work has been performed to incorporate this predator into integrated pest management programs in maize [22]–[26].

The invasion of *H. axyridis* in Brazil may lead to increased exposure of *S. frugiperda* to this new predator in different cropping systems. However, we are not aware of previous predation studies of *H. axyridis* preying on *S. frugiperda.* The objective of this study was to determine if an important generalist predator, *H. axyridis,* now occurring in North and South American areas of maize production, displays a preference between prey larvae fed GE maize (Bt) or conventional maize (non-Bt), which could in turn potentially impact resistance development.

## Results

**Figure 1:**
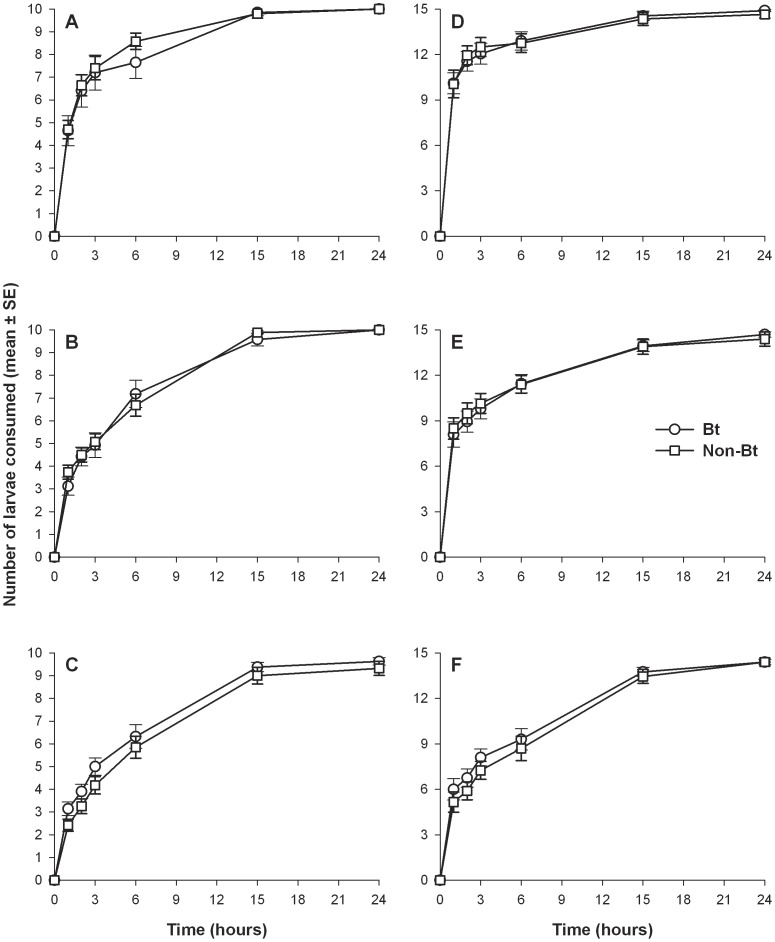
Number of Bt-fed and non-Bt-fed *Spodoptera frugiperda* larvae consumed over time by *Harmonia axyridis*. Predator third instars (A, B, C) and adult females (D, E, F). Prey larvae were 24-h-old (A, D), 48-h-old (B, E), and 72-h-old (C, F).

The *S. frugiperda* larvae that fed on non-Bt maize leaves were significantly larger than those fed Bt leaves. The 24-h-old prey larvae fed non-Bt maize weighed 0.0041±0.000068 g (mean±SE) and those fed Bt maize weighed 0.0038±0.00004 g (t = −14.03, df = 8, p = 0.0001), 48-h old prey fed non-Bt weighed 0.0069±0.00075 g and those fed Bt weighed 0.0047±0.00016 g (t = −2.92, df = 8, p = 0.0139), and 72-h-old prey fed non-Bt weighed 0.0073±0.00033 g and those fed non-Bt 0.0041±0.000097 g (t = −9.44, df = 8, p = 0.0001). Despite the differences in larval size, no significant differences or consistent numeric trends were found between the numbers of prey consumed for Bt and non-Bt fed larvae for any of the predator stage by prey age combinations (Fig. 1; Table 1). However, more time appears to have been required for *H. axyridis* adults to consume older larvae (i.e., 72-h-old). By the end of the experiment with 24- and 48-h-old larvae all third instar *H. axyridis* ate 20 available prey and with 72-h-old larvae the predator consumed an average (±SE) of 19.27±0.51; whereas the female adult *H. axyridis* did not consume all of 30 prey available (an average of 29.55±0.24, 29.1±0.46 or 28.8±0.38 larvae consumed of 24-, 48- or 72-h-old larvae, respectively).

**Table 1:** Test statistics of prey preference for *Harmonia axyridis* life stages preying on three ages of *Spodoptera frugiperda* larvae fed Bt versus non-Bt maize at 3 and 6 h after the beginning of experiments.

	3 h			6 h		
Experiment	S*	p	N	S*	p	N
A	−0.5	0.982	20	−7.5	0.250	14
B	−9	0.822	26	19.5	0.256	16
C	54	0.169	29	13	0.519	19
D	−7	0.564	20	3.5	0.699	20
E	−13.5	0.625	20	1.5	0.960	20
F	31.5	0.074	20	22	0.265	20

* Wilcoxon signed rank test.

Marking of prey larvae did not significantly affect prey preference for predators exposed to 24-h-old prey (S = 5.5, p = 0.3750), 48-h-old prey (S = −2.0, p = 0.8828) and 72-h-old prey (S = −3.5, p = 0.5000). Furthermore, in arenas with Bt-fed and non-Bt-fed prey and no predator, overall rates of cannibalism were low (0.25±0.10, 0.47±0.15, 0.54±0.13 larvae consumed for 24-, 48-, and 72-h-old prey, respectively) and did not differ significantly between Bt-fed and non-Bt-fed prey that were 24-h-old (S = −6, p = 0.5625), 48-h-old (S = 2, p = 1) and 72-h-old (S = 0, p = 1). Therefore, neither marking nor the rates of cannibalism were likely to have significantly influenced the results.

Bt maize leaves contained mean Cry1Ab concentrations of 23–33 µg/g dry weight (Table 2). There was no decrease in Bt protein detection up to 72 h after the leaves were cut from the plants. Larvae of *S. frugiperda* feeding on Bt maize for 24 to 72 h contained one order of magnitude less Cry1Ab than maize leaves (2.1–2.2 µg/g dry weight). The values for the different feeding times were very similar. Third instar larvae and females of *H. axyridis* preying for 24 h on caterpillars from Bt and non-Bt maize in the choice assays contained mean concentrations of 0.05–0.17 and 0.01–0.02 µg Cry1Ab/g dry weight, respectively. Those values were near to the LOD, which was 0.03 and 0.007 µg/g dry weight for *H. axyridis* larvae and females, respectively. No Cry1Ab was detected in non-Bt leaves (LOD = 0.01 µg/g dry weight) and prey larvae feeding on non-Bt leaves (LOD = 0.03 µg/g dry weight).

**Table 2:** Cry1Ab concentrations in µg/g dry weight of maize leaves (event MON810, DKC-5048 RR2) measured at 0, 24, 48, and 72 hours after initiation of experiments, *Spodoptera frugiperda* larvae allowed to feed on Bt maize leaves for 24 h, 48 h, or 72 h, and *Harmonia axyridis* larvae (3rd instar) and females having the choice between Bt and non-Bt maize fed *S. frugiperda.*

Time point	Maize leaves	*S. frugiperda*	*H. axyridis*		Experiment
**0 h**	24.9±1.24 (N = 10)				
**24 h**	23.4±2.35 (N = 10)	2.1±0.29 (N = 5)	0.05±0.014 (N = 10)	3^rd^ instar	A
			0.02±0.005 (N = 10)	Female	D
**48 h**	30.5±1.36 (N = 10)	2.2±0.16 (N = 5)	0.10±0.041 (N = 10)	3^rd^ instar	B
			0.01±0.002 (N = 10)	Female	E
**72 h**	33.4±2.13 (N = 10)	2.1±0.34 (N = 5)	0.17±0.040 (N = 10)	3^rd^ instar	C
			0.01±0.004 (N = 20)	Female	F

Non-Bt maize leaves: < LOD (0.01 µg/g DW).

Non-Bt *S. frugiperda*: < LOD (0.03 µg/g DW).

## Discussion

With the worldwide range of *H. axyridis* expanding, particularly in South America [18], [19], the likelihood of this predator co-occurring with new prey will continue to increase. An abundant potential prey item for the lady beetle is *S. frugiperda* an economically damaging pest of maize and cotton in Brazil and South America [4]–[6], [27], [28]. The data presented here indicate that larvae and adults of *H. axyridis* will prey on 24- to 72-hour-old *S. frugiperda* larvae under laboratory conditions. Though considered to be primarily an aphidophagous predator, *H. axyridis* has been documented preying on eggs and larvae of several lepidopteran species [21]. In functional response studies, adults and larvae of *H. axyridis* consumed 15 first instar larvae of *Danaus plexippus* L. (Lepidoptera: Danaidae) per day [29]. Overlap of the phenology of this predator and prey species in the field should be examined along with in field predation rates to more thoroughly assess the level of natural pest suppression this predator may offer in newly invaded areas.

Furthermore, this study examined whether *H. axyridis* shows a preference between Bt-fed and non-Bt-fed *S. frugiperda* larvae. The potential risk of Lepidopteran-resistant Bt crops to coccinellids and other predators has received considerable attention [30]–[32]. Studies on coccinellids have examined population level responses in the field [33]–[36]. Furthermore, potential direct impacts of Lepidoptera-active Bt Cry toxins to coccinellids that consume purified toxin or Bt plant tissue have been studied [10], [37]–[40]. In contrast, relatively little work has been conducted to examine predator preference for prey fed Bt versus non-Bt plant tissue [41], [42].

In the present study, a sublethal effect of Cry1Ab on *S. frugiperda* larvae was apparent with larvae that were fed Bt maize being significantly smaller than those fed non-Bt maize. This finding confirms earlier reports for *S. frugiperda* [7], [43] and the closely related *Spodoptera littoralis* (Boisduval) [44], [45].

Bt maize in our study was quantified, expressing 23–33 µg Cry1Ab/g dry weight, which corresponds to 4.1–4.6 µg/g fresh weight. These values are comparable to published data from the field [46]. Larvae of *S. frugiperda* feeding on Bt maize contained an order of magnitude of lower concentrations of Cry1Ab than the leaves. Furthermore, lady beetle larvae and female beetles, which consumed both prey from Bt and non-Bt leaves, contained only traces of the Bt protein. This confirms that Bt proteins are highly diluted along the food chain. Comparable dilution levels have been reported from other laboratory studies using tri-trophic systems of Bt plants, lepidopteran larvae and coccinellid predators [10], [40], [47].

Feeding time on Bt maize apparently has no influence on Bt concentrations in *S. frugiperda* larvae (Table 2). However, lady beetle larvae contained more Cry1Ab when feeding on caterpillars that were kept longer on maize leaves (0.05, 0.10, 0.17 µg/g dry weight for 24, 48, and 72 h, respectively). One potential explanation could be that the larger size of older larvae allowed the lady beetle to ingest more Bt protein. This, however, was not observed for female lady beetles. Nevertheless, preference for larger insects was not tested as we did not have access to a Cry1Ab resistant colony of *S. frugiperda*.

The protein Cry1Ab concentrations in 4^th^ or 6^th^ instar *S. frugiperda* larvae were approximately 40% of the Cry1Ab concentration found in the Bt maize that was consumed [43]. Relatively high levels of Cry1Ab toxin have also been found in *S. littoralis,* where larvae feeding for 8 or 11 days on MON810 maize leaves contained approximately one third of the Cry1Ab concentration in the leaves [45]. On the other hand, neonate *O. nubilalis*, *Agrotis ipsilon* (Hufnagel) (Lepidoptera: Noctuidae), and *Helicoverpa zea* (Boddie) (Lepidoptera: Noctuidae) feeding on MON810 maize of the same age as in the present study contained only 1–2% of the concentration in the corresponding leaves [48].

Despite sublethal effects of Bt maize on prey size and the presence of Bt toxins in Bt-fed prey, *H. axyridis* showed no preference between Bt-fed and non-Bt fed prey. In all combinations of predator stage and prey age, the number of each prey type (i.e., Bt-fed and non-Bt-fed) consumed did not differ significantly. In a similar study, *Chrysoperla carnea* (Stephens) (Neuroptera: Chrysopidae) third instars could choose between *S. littoralis* fed Bt maize and *S. littoralis* fed non-Bt maize, the lacewing larvae showed a preference for *S. littoralis* fed non-Bt maize; however, *C. carnea* first and second instars showed no preference. In addition, *C. carnea* first to third instars did not show a preference between *Rhopalosiphum padi* (Homoptera: Aphidae) fed-Bt and non-Bt maize [41]. In addition, *Poecilus cupreus* L. (Coleoptera: Carabidae) ingested *S. littoralis* larvae readily and did not appear to avoid Bt maize-fed prey [42].

Seed mixtures (“refuge in a bag”), which result in non-Bt plants being interspersed in Bt fields, are currently being promoted for insect resistance management (IRM). The resulting mixture of non-Bt plants with Bt plants will result in predators being confronted with a choice between Bt-fed and non-Bt-fed prey. If a predator shows preference for a specific type of prey the rates of resistance development could be impacted [15]. For example, if a predator prefers to feed on herbivores that fed upon non-Bt plants, then that predator might kill a disproportionate fraction of the relatively few non-Bt exposed larvae that developed on the non-Bt refuge plants in the field, which could affect rates of resistance development. The results of the present study suggest that such a behavioral mechanism to altering rates of resistance development are not likely to exist for *S. frugiperda* with *H. axyridis* as a main predator, but this interaction should be investigated for other lepidopteran herbivores. Also, predator responses to varying densities of prey on Bt and non-Bt plants have not been investigated in the current study, but could become important in the field.

## Materials and Methods

### Plant Material

Two maize hybrids were utilized in this study: a GE hybrid, DKC-5048 RR2 (Bt) (event MON810), and conventional near isoline, DKC-4840 (non-Bt), both from Monsanto Company. The DKC-5048 RR2 plants express the *cry1Ab* gene from Bt, targeting lepidopteran pests. The plants of both hybrids were grown under the same environmental conditions of 27±1°C, 70±5% RH, and 16∶8 (L: D) h cycle, in a greenhouse. Seeds were sown weekly in one-liter pots (4 seeds per pot) filled with Metromix 582 (Sun Gro Horticulture, Bellevue, WA) potting soil. Leaf tissue was taken from plants between V3 and V6 growth stages [49] to feed *S. frugiperda* larvae.

Plant material was taken for Cry1Ab quantification. The samples were obtained from a middle-upper leaf of a Bt or non-Bt plant. The leaves were cut, put in a plastic containers (18 cm×8 cm) in a growth chamber, and sampled at 0, 24, 48, or 72 h after the being put in that container. Each sample consisted of one leaf piece, 1 cm in diameter. Ten samples from different Bt plants and five from non-Bt maize plants were taken for each time point. All samples were weighed and placed into separate 1.5-ml centrifuge tubes for each sample, and kept at −80°C for Cry protein measurement using ELISA.

### Insect Material

Eggs of *S. frugiperda* were purchased from French Agricultural Research (Lamberton, MN). Upon arrival, egg masses were placed in plastic containers (18 cm×8 cm) and kept in a growth chamber at 25±1°C, 70±5% RH, and 16∶8 (L: D) h cycle until eclosion. The larvae were given an ad libitum supply of maize leaves for either 24, 48 or 72 h. The same leaves remained with the larvae for these respective periods of time. A subset of larvae was used for determining larval mass for each feeding treatment by age group combination. A balance (Mettler AE260 DeltaRange®) was used to weigh groups of larvae (Bt-fed: 5 groups of 50 24-h-old larvae, 5 groups of 40 48-h-old larvae, 5 groups of 20 72-h-old larvae; non-Bt-fed: 5 groups of 50 24-h-old larvae, 5 groups of 40 48-h-old larvae, 5 groups of 20 72-h-old larvae), from which mean individual larval weights were calculated.

Third instar larvae and female adults of *H. axyridis,* were obtained from a laboratory colony maintained at the University of Minnesota. Eggs of *H. axyridis* were placed into Petri dishes (10×1.5 cm) in a growth chamber at 25±1°C, 70±5% RH with a 16∶8 (L: D) h cycle. Twenty-four hours after eclosion, first instar *H. axyridis* were placed individually in 6×1.5 cm Petri dishes, and reared with an ad libitum supply of *Ephestia kuehniella* (Zeller) (Lepidoptera: Pyralidae) eggs. Water was provided via moistened florists foam. The predators were reared under these conditions to the desired life stages (i.e., 6 to 24 h-old third instars and 6 to 48 h-old adult females) for use in the bioassay. All *H. axyridis* were starved for 24 h prior to initiation of the experiments.

Prey (*S. frugiperda*) and predator (*H. axyridis*) were also assayed for Bt protein content. Five samples of larvae feeding on Bt and non-Bt maize were taken from each prey age group (24, 48 and 72 h-old larvae). Each sample contained approximately 30 larvae. For the predator, 10 samples were taken from third instars that fed on 24, 48 and 72 h-old prey larvae; also 10 samples from adult females that fed on 24 and 48 h-old larvae and 20 samples from females that fed on 72 h-old larvae. Each predator sample contained one specimen. All the insect material was collected and stored in 1.5-ml centrifuge tubes at −80°C until the ELISA assays were run.

### Choice Tests

Petri dishes (6×1.5 cm) were used as experimental arenas and the experiments were conducted in a climate controlled chamber at 25±1°C, 70±5% RH with a 16∶8 (L: D) h cycle. For the study of choice behavior, third instar and adult females of *H. axyridis*, were offered 24, 48 or 72 h-old larvae of each prey type (i.e., *S. frugiperda* fed Bt maize and *S. frugiperda* fed non-Bt maize) as prey. The combinations of predator stages and prey treatments are summarized in Table 3. Third instar *H. axyridis* received 10 prey items of each type and adult *H. axyridis* received 15 prey items of each type, resulting in a total of 20 or 30 prey items for a single predator larva or adult per arena, respectively. Petri dish arenas were sealed with parafilm to prevent escape of small larvae. To track which prey fed on Bt or non-Bt maize, prey of one treatment group were marked on the dorsal surface of the abdomen with a red permanent marker (Sharpie Ultra-Fine Point Permanent Marker, Newell Rubbermaid Office Products) just before the beginning of each observation. Marking of Bt or non-Bt fed prey was randomized among arenas to account for any potential effects of the marking. Each of the 6 choice test experiments was conducted separately, with 14 to 29 replications per experiment (Table 3). The number of replications varied in each experiment because larvae either molted to the next instar during the trial and were therefore removed from the trial, or some observation intervals were not included (e.g., 6 h) in all replications. Visual observations of arenas were conducted at 1, 2, 3, 6, 15 and 24 h after the start of the experiment to determine the number and type (i.e., Bt-fed and non-Bt-fed) of prey consumed by each individual *H. axyridis.*

**Table 3:** Treatment combinations tested in the choice-test experiments. *Harmonia axyridis* larvae or adults were given the choice of Bt and non-Bt-fed *Spodoptera frugiperda* larvae.

Experiment	Predator	Prey choice 1	Prey choice 2
A	3^rd^ instar	24 h-old larvae fed non- Bt corn	24 h-old larvae fed Bt corn
B	3^rd^ instar	48 h-old larvae fed non- Bt corn	48 h-old larvae fed Bt corn
C	3^rd^ instar	72 h-old larvae fed non- Bt corn	72 h-old larvae fed Bt corn
D	Adult female	24 h-old larvae fed non- Bt corn	24 h-old larvae fed Bt corn
E	Adult female	48 h-old larvae fed non- Bt corn	24 h-old larvae fed Bt corn
F	Adult female	72 h-old larvae fed non- Bt corn	24 h-old larvae fed Bt corn

In addition to the above mentioned randomization of the marking of Bt and non-Bt-fed prey, the potential influence of the permanent marker on prey preference was investigated in separate trials carried out prior to the experiments. In each Petri dish arena there were 10 marked and 10 unmarked larvae that were fed with the same type of maize, either Bt or non-Bt. This test was conducted with third instar *H. axyridis,* which were offered prey that was 24 and 48 h old (10 replicates each), and prey that was 72 h old (7 replicates). Petri dish arenas and experimental conditions were as described above.

To assess the potential for cannibalism between Bt-fed and non-Bt-fed prey, experiments were conducted with only prey in the arena. For these cannibalism experiments, 10 Bt-fed larvae and 10 non-Bt larvae were placed in Petri dish arenas. The cannibalism experiment was conducted with the same time treatments than the choice test, i.e. with 24 h (25 replicates), 48 h (15 replicates) and 72 h (10 replicates) old larvae. Prey were marked as described above and the number and type of prey consumed by other larvae was recorded. Petri dish arenas and conditions were as described above.

### Cry1Ab Quantification using ELISA

Samples of leaves, *S. frugiperda* and *H. axyridis* were lyophilized and the dry weight (DW) was determined. Leaf samples were cut into small pieces using a pair of scissors. One tungsten carbide ball (5 mm) was added to each microreaction tube together with phosphate-buffered saline with Tween (PBST) at a volume of 300 µl for insect samples and 500–750 µl for leaf samples. The samples were homogenized for 3 min at 30 Hz in a Tissue Lyser II (Quiagen, Hombrechtikon, Switzerland). After centrifugation at 13000×g for 3 min, the supernatants were diluted with PBST 100 times for Bt maize leaves and 5 times for *S. frugiperda* samples. Non-Bt samples and *H. axyridis* samples were used undiluted. The concentrations of Cry1Ab were measured using double-antibody sandwich enzyme-linked immunosorbent assays (DAS-ELISA) commercially available from Agdia (Elkhart, IN). To be able to use the kit for quantitative measurements, we prepared six concentrations of Cry1Ab solution between 0.3 and 10 ng/ml for building a standard curve. Purified Cry1Ab of attested purity and quality was provided by Monsanto. The plates were processed according to the manufacturer’s protocol. The optical density (OD) was measured at 620nm light wavelength with a SpectrafluorPlus plate reader (Tecan, Männedorf, Switzerland). Cry1Ab concentrations (in µg/g dry weight) were calculated from the standard curve using the regression analysis. The limit of detection (LOD) of the test system was determined based on the standard deviation of the OD values of all non-Bt samples multiplied by 3.

### Data Analysis

Analyses were performed using the SAS statistical package [50]. For each prey age class, mean larval masses of Bt and non-Bt-fed individuals were compared using a two sample *t*-test (PROC TTEST). For each prey age class and predator stage combination of the choice test experiment, the number of Bt-fed versus non-Bt-fed prey consumed was compared using the Wilcoxon signed rank test (PROC UNIVARIATE). As the consumption data were cumulative during the analyzed time, we performed the statistical analyses considering just two observation points from each bioassay. The 3 and 6 hour observation points allowed enough time for most predation to occur yet was prior to the plateau in prey consumption. A similar Wilcoxon signed rank test was conducted for the cannibalism and marking studies, but only for observations at 6 h.
